# The Current Status, Prospects, and Challenges of Shape Memory Polymers Application in Bone Tissue Engineering

**DOI:** 10.3390/polym15030556

**Published:** 2023-01-21

**Authors:** Tingting Li, Liang Chen, Yu Yuan, Rengfei Shi

**Affiliations:** 1 School of Exercise and Health, Shanghai University of Sport, Shanghai 200438, China; 2 School of Exercise and Health, Guangzhou Sport University, Guangzhou 510500, China

**Keywords:** shape memory polymers, scaffolds, bone tissue engineering, bone defects

## Abstract

Bone defects can occur after severe trauma, infection, or bone tumor resection surgery, which requires grafting to repair the defect when it reaches a critical size, as the bone’s self-healing ability is insufficient to complete the bone repair. Natural bone grafts or artificial bone grafts, such as bioceramics, are currently used in bone tissue engineering, but the low availability of bone and high cost limit these treatments. Therefore, shape memory polymers (SMPs), which combine biocompatibility, biodegradability, mechanical properties, shape tunability, ease of access, and minimally invasive implantation, have received attention in bone tissue engineering in recent years. Here, we reviewed the various excellent properties of SMPs and their contribution to bone formation in experiments at the cellular and animal levels, respectively, especially for the repair of defects in craniomaxillofacial (CMF) and limb bones, to provide new ideas for the application of these new SMPs in bone tissue engineering.

## 1. Introduction

With the advancement of bone biology research and material engineering, the materials used for bone defect repair in bone tissue engineering are constantly being updated [1]. Current treatments of critical bone defects include natural bone grafts such as autologous bone grafts, allogeneic bone grafts, allograft bone grafts, and artificial bone implants such as metal and bioceramics, which suffer from the problems of adding additional wounds, low availability, immune rejection, slow curing times. Furthermore, metal for internal implantation and external fixation is often required when faced with complex bone defects [2,3]. However, these metal implants or external fixators can lead to delayed or malformed fracture healing or even non-healing due to their non-degradable nature, the stress shielding effect, and restricted angiogenesis [4,5,6]. Biopolymers such as injectable hydrogels have shown some advantages in repairing bone defects, such as minimally invasive implantation and filling irregular defects. However, their utilization in bone tissue engineering is limited by their lack of porous structure and mechanical properties that are insufficient to meet the load-bearing requirements of bone tissue or the high requirements for scaffold fabrication due to their non-adjustable shape after fabrication [7,8].

As a result, bone tissue engineering-related projects began to search for a new material that could simultaneously ameliorate these problems, and shape memory polymers (SMPs) were noted for their unique biocompatibility, biodegradability, mechanical properties, and minimized invasiveness. Especially for their shape tunability, which holds promise for treating irregular bone defects. SMPs undergo a shape transition between a temporary shape and a permanent shape when stimulated by thermal [9], water [10], magnetic [11], and ultrasonic [12]. We describe this reversible transformation between different shapes as the shape memory effect (SME), which mainly originates from the two dual-phase structures, the fixed phase, and the reversible phase. The fixed phase is designed to maintain and restore the original shape and prevent flow deformation of the polymer, while the reversible phase is to fix the temporarily deformed shape. For example, thermosensitive SMPs can be deformed into the desired temporarily deformed shape upon heating. The temporary shape can be fixed upon cooling. When reheated, the temporarily deformed shape returns to its original permanent shape and is accompanied by a constant change in stiffness [13]. The emergence of SMP materials has attracted widespread interest in the biomedical sciences, with cardiovascular stents [9], self-tightening sutures [14], dialysis needle adapters [15], and thrombus removal devices [16] all focusing on SMPs and resulting in satisfactory clinical outcomes [13].

Based on the successful demonstration of SMPs in various tissue engineering, scientists have developed a strong expectation of whether they could also play a role in bone tissue engineering. In addition to the advantages of minimally invasive implantation, the expansion forces generated by the shape restoration activation of SMPs can also provide good self-adaptation in bone defects, with the expanded dense SMPs matching the borders of the bone defect perfectly [17]. The resulting constraint between this bone defect and the scaffold benefits bone growth [18]. Existing studies have used SMPs in various cellular experiments and animal models of bone defects to initially validate the potential use of SMPs. However, SMPs are not yet fully adequate for repairing all types of bone defects due to the stringent requirements of bone tissue engineering for various properties of implant materials, such as complex bone defects in the load-bearing bone. Therefore, this study reviewed the current status, prospects, and challenges of the application of SMPs in bone tissue engineering, aiming further to elucidate the role of SMPs for bone defect repair and therefore provide a theoretical basis for the application of SMPs in bone tissue engineering and to provide new directions for the development of new smart materials in bone tissue engineering.

## 2. The Primary Materials of SMPs and Their Synthesis

SMPs are intelligent materials with a wide range of sources. At present, the typical raw materials include polycaprolactone (PCL), polylactic acid (PLA), polyurethane (PU), and the semiconductor poly (L-lactide) (PLLA). They are used to synthesize therapeutic scaffolds or carriers for tissue engineering through photochemical solidification (SCPL), electrospinning, and gas foaming [19,20,21,22]. Additionally, the material is frequently immersed in granular, molten salt, or sugar when making SMP scaffolds. After curing, these particles are evenly distributed throughout the SMPs material, and the material is soaked in the solution. The porous SMP scaffold is created after the particles are melted and dried. Shape memory polyurethane, physically crosslinked SMP scaffolds, and chemically crosslinked SMP scaffolds are different materials that can be used to prepare SMP scaffolders (SMPU). According to how their shape is recovered, they can be classified as self-deploying scaffolds, self-expanding scaffolds, and self-fitting scaffolds. The self-deploying scaffolds are usually implanted in the body via a minimally invasive form with a transition temperature (T_trans_) close to body temperature (Tbody~37 °C) to trigger shape recovery, and the self-expanding scaffolds and self-fitting scaffolds are usually implanted in the defect area after being triggered by temperature or water in vitro rather than minimally invasive implantation. Among them, the T_trans_ of self-fitting scaffolds is usually slightly higher than Tbody, which can be heated in an environment such as normal saline to cause softening or activate shape memory function through hydration, thus allowing the scaffolds to be pressed into tissue defects. The shape recovery drive bracket expands within the defect, including those with irregular geometry. When the scaffold is cooled to ~37 °C, it becomes a shape fixed in this new geometry [13].

A single-shape memory material usually cannot meet the clinical multi-property needs of biomedical applications. For example, some primary SMP scaffolds that can be made from PCL by foaming or SCPL suffer from inadequate biocompatibility, low degradation rates, and insufficient mechanical rigidity, which significantly limit the effective application of PCL [22,23,24]. Therefore, a great deal of work on the biomedical applications of PCL (SMPs) is trying to increase their toughness and SME by blending with other polymers, just as PCL optimizes its properties by combining with poly(propylene carbonate) (PPC), 1,2,4-triazoline-3,5-dione (TAD), and PLLA [25,26,27]. In recent years, with the continuous progress of biochemical materials research and development technology and due to the high availability and minimized invasiveness of SMP scaffolds, many studies have focused on improving the therapeutic potential of SMP scaffolds in various diseases [28]. However, SMP scaffolds have different property requirements in practical applications. Therefore, SMP scaffolds that can respond to different stimulation come into being.

## 3. SMPs and Their Properties in Response to Different Stimulations

**Figure 1:**
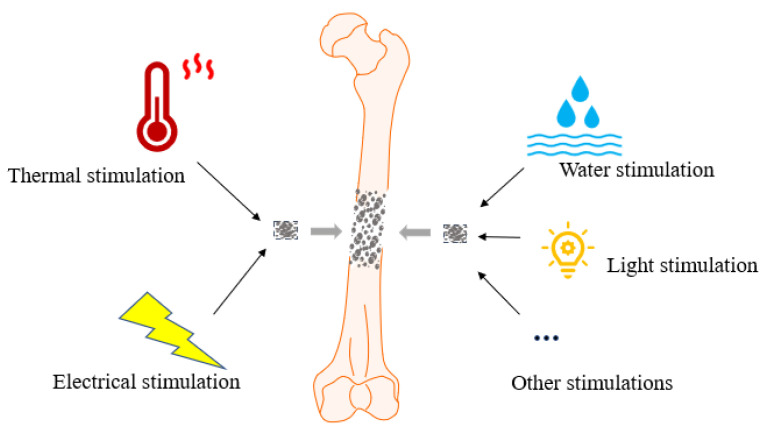
Schematic diagram of SMPs applied to bone tissue engineering after various stimulations.

SMPs are mainly divided into thermology-responsive and water-responsive materials, light-responsive materials (ultraviolet), and electro-responsive materials [29,30,31,32]. The shapes can be deformed and restored under activation by thermal, water, light, electrical and magnetic stimuli, thus performing their properties effectively (as shown in Figure 1). And they are suitable for the repair and regeneration of different types of tissue since each material has its advantages and disadvantages.

### 3.1. SMPs in Response to Different Stimulations

#### 3.1.1. Thermology-Responsive SMPs

Most SMPs used in tissue engineering are thermology-responsive materials that can be activated by a thermal source giving a critical transition temperature. The principle is that when an external temperature stimulates the SMP scaffold, the material changes into any desired permanent shape. The thermal response SMPs have a relatively crucial thermal performance, mainly involving two types. One type is close to the physiological temperature. Guo et al. found that the SMP polymer made of PLCL and poly(l-lactide-*co*-glycolide) (PLGA) has a shape recovery rate of up to 100% when the temperature is close to the human body temperature, and the SMP material shows high elasticity at 37 °C [33]. The second type is higher than the physiological temperature. For example, the transition temperature of the thermology-responsive material made of PLA is 59 °C, and the dissolution temperature is 150 °C, which often causes significant damage to human tissues or cells [29]. Although thermology-responsive SMPs are the most widely used, they still have some shortcomings because they are essentially affected by temperature, and the human body temperature is only about 37 °C. When the temperature affecting the shape memory polymer is higher than the body temperature, the activity of human cells and tissues will be affected to some extent. In some special clinical applications, it is unsuitable for materials with high elasticity and rapid shape and thermal sensitivity recovery. Thermally responsive SMPS also have disadvantages, such as uneven internal temperature distribution and different surface and internal temperature transfer rates [34]. Therefore, other types of SMPs gradually become a new research hotspot.

#### 3.1.2. Water-Responsive SMPs

Water-responsive SMPs scaffolds are hydrophilic fabrication materials and can be plasticized or driven by water in an aqueous environment to undergo shape transformation [30]. They can be made from a composite of L-lysine diisocyanate (LDI), polyethylene propylene (PHA), and polyethylene glycol (PEG). When Wang [35] used a 3D elastic scaffold made of LDI, PHA, and PEG for water-response SMP, they found that the scaffold quickly recovered to 90% shape when placed in water. It has been found that water-responsive SMPs have fewer restrictions compared to thermology-responsive SMPs. For example, SMP scaffolds made from water-responsive poly(butanetetrol fumarate) (PBF) can compensate for the limitations of using other materials due to harmful effects on tissues such as temperature, UV, and electricity. Moreover, its unique advantages are its inherent biocompatibility and the off-the-shelf availability of PBF scaffolds. In conclusion, water-responsive SMPs have become the new research hotspots in tissue engineering for their short time, high efficiency, and gentleness of shape recovery ability [36]. However, some water-responsive SMPs were found to be very slow in their response to water for more than a month due to their hydrophobic fabrication material and lack of modification sites to modulate their properties resulting in them being biologically inert [30]. For these reasons, developing new water-responsive switching power supplies for biomedical applications is highly desirable.

#### 3.1.3. Electro-Responsive SMPs

With the widespread use of SMPs in recent years and the emergence of the need for remote control, electrically sensitive SMPs with electrical actuation capabilities have been developed, which are polymers activated by voltage or electrical stimulation and have a shape memory effect, usually prepared from materials such as graphene, carbon nanotubes (CNTS) and metals [37,38], using a conductive shape memory material that combines polypyrrole (PPy) and PLA. When low voltage stimulation was applied to the material, it was found that it could restore its original shape within 2 s and would not produce thermal inequality such as thermology-responsive materials. In short, it had the characteristics of polylactic acid materials and could be stimulated by electricity [31]. However, current research on SMPs of electrically sensitive materials is not sufficiently advanced, as the techniques for remote control of electrical stimulation are not yet well developed.

#### 3.1.4. Light-Responsive SMPs

In addition to the above SMPs, light-responsive SMPs have also been recently investigated in tissue engineering. Compared with thermology-responsive SMPs and water-responsive SMPs, light-responsive SMPs have advantages such as controllable space, long-distance propagation, and more free movement of molecules [32]. Photosensitive materials are generally divided into two types, one is the photosensitive materials made of their materials, and the other is the light-responsive SMPs combined with other types of SMPs to produce a common effect, such as the addition of photothermal filler in the thermology-responsive SMPs to produce photothermal effect [39]. Simple photosensitive material polymers, such as Xu [40], used maleated polylefin elastomer (mPOE)/polyaniline (PANI) to prepare photosensitive polymers. After irradiating the surface with infrared (1.0 W/cm), they found that mPOE and PANI formed crosslinking and would change with the increase of PANI content and returns to their original shape when exposed to NIR light.

Various stimulus-responsive SMPs have significantly been used in the biomedical field because of their different advantages. However, single stimulus-responsive SMPs usually have a limited scope of application, so integrating various SMP materials may have more significant advantages.

### 3.2. Properties of SMPs

#### 3.2.1. Biocompatibility and Biodegradability of SMPs

The performance of SMPs determines whether SMP materials can be used in biology and clinic. Shape memory properties mainly include biocompatibility, biodegradation, and mechanical properties [41]. Many studies have shown that PCL has certain biocompatibility and degradability [23,42], in which the porous structure makes a specific contribution because appropriate pore size is conducive to the invasion and growth of cells and tissues into the scaffold, and finally, the completion of regeneration and repair. The size of molten salt or sugar particles directly determines the pore size of the porous structure in SMPs. For example, Wang [43] synthesized shape memory polymers with a pore size of 500 μm using the sugar particle method, which was found to be a suitable space for tissue and cell growth. Moreover, the biological properties of SMPs will be reduced when their pore size is too small. Research has found that polydopamine (PD) coating provides an additional biological activity to biological materials as a substrate attachment. Therefore, research is currently testing dopamine after coating SMP scaffolds with PD [24]. In addition, the biocompatibility of SMPs can be derived from the unique functional structure of its materials. For example, the components, including fumaric acid and butatetraol in PBF material, have good biocompatibility. In addition, some SMP materials can also promote cell adhesion and proliferation due to the abundance of hydroxyl and carboxyl groups. In most studies, there is a correlation between the biocompatibility and biodegradability of SMPs.

In biology, hydrolysis and oxidation are often used as degradation mechanisms for SMPs, while biodegradable biomaterials usually have enzyme bonds and hydrolyzability [44]. In addition, SMPs can be degraded in the process of bone regeneration, avoiding the second surgery to remove the metal fixation and not worrying about the service life limitation like other biopolymeric materials. Therefore, SMPs are often used in clinical applications through hydrolysis and oxidation.

#### 3.2.2. Mechanical Properties of SMPs

Biological properties cannot wholly judge the ability of SMPs, and most studies will combine the ductility, tenseness, high elasticity, and strain ability of its physical properties to evaluate the applicability of an SMP. Paderni [45] used water-induced PBS-silica networks to prepare silane crosslinks and found that the elasticity of rubber modulus would gradually increase as the network density and silica content increased. Furthermore, a new grid-like SMPs composed of 80% polyethylene glycolamino (PEGA) and 20% tert-butyl acrylate (TBA) formula was found to have good rigidity and high tensile strength after dynamic mechanical tests and tensile tests [46]. SMP, made from a mixture of acrylate-based and polystyrene (PS), exhibits excellent ductility and viscosity [47]. Moreover, their elaboration of liquid crystal polymers mentions that this unique reversible shape polymer has large-scale and high-speed actuation [48]. The PCL and PU blend is rigid and flexible [49]. Moreover, the tensile strength and elasticity of the SMP scaffold are gradually improved; the tensile strength of SMP is 48 MPa, the flexural strength is 55 Mpa, and the tensile strength and flexural strength of SMP formed by 3D printing by particle extrusion are 55.58 and 73.4, respectively, and the tensile strength and modulus of SMPs prepared by ethyl cellulose (EC) and PCL are as high as 100 Mpa. It is sufficient to support soft tissue engineering applications, and the porous structure prepared with SMP is more sensitive and durable than other materials [19,50].

In short, biocompatibility, degradability, and mechanical properties are the most distinctive properties of SMP materials. The fact that these properties can also be adapted to requirements in practical applications has led to their extensive research in various fields. In addition, these scaffolds’ sterility depends mainly on prolonged UV irradiation or autoclaving. At the same time, recent studies have shown that the antimicrobial properties of the scaffolds themselves can be improved by adding additional antimicrobial components such as Fe_3_O_4_ [51,52], which will further enhance the application of SMP scaffolds in clinical tissue engineering if antimicrobial properties are obtained.

## 4. Application of SMP Scaffold in Bone Tissue Engineering

SMPs have emerged in research since the 1980s. They have been widely used in aerospace engineering and biomedical devices after continuous optimization. They have caused more and more concerns for scientists in bone tissue engineering due to their excellent performance [53,54]. Since 2014, various studies have repeatedly tested the performance of various SMP scaffolds according to the needs of bone tissue regeneration and repair properties. Currently, studies are gradually tapping the maximum potential of SMP scaffolds in bone tissue engineering in vitro and in vivo experiments.

### 4.1. Osteogenic Properties of Bone Formation-Associated Cells in SMPs

The evaluation of the effectiveness and reliability of the treatment modality in bone tissue engineering is mainly to detect whether the new bone is formed in the defect. Bone formation is mainly mediated by osteoblasts, MC3T3-E1, and C2C12 myogenic cells as well as some stem cells can also differentiate into osteoblasts under osteogenic induction conditions, such as bone marrow mesenchymal stem cells (BMSCs), adipose stem cells (ASCs). Therefore, all these cells were used in bone tissue engineering to initially validate the role of SMP composed of different materials on osteogenic differentiation.

#### 4.1.1. Effect of SMPs on Osteoblasts and the Osteoblast Cell Line MC3T3-E1

In the early stages of studying the potential of SMP in bone tissue engineering, rat and human osteoblasts and MC3T3-E1 were used to observe the effects of SMP on cell adhesion, proliferation, and differentiation ability [17,55,56]. Based on the shape memory advantages of poly (D,L-lactide) (DLLA) in biomedicine, Zhang et al. [55] synthesized poly (D,L-lactide-*co*-trimethylene carbonate) (referred to as PDLLA-*co*-TMC or PLMC) by electrospinning a copolymer of D,L-propyleneglycolate (DLLA) monomer and trimethylene carbonate (TMC) monomer. The glass transition temperature (T(g)), mechanical properties, and degradation properties of PLMC can be varied with the change of the ratio of the two monomers, so the T(g) of PLMC can be adjusted to be close to the body temperature to accommodate the shape memory in the human body. It was found that PLMC can recover the shape in 10 s at 39 °C, and the PLMC synthesized with different ratios of DLLA and TMC in the study had a porosity of 71.8–79.3% and a pore size of 12.4–19.7 μm. Functionally, they had considerable cytocompatibility to support the adhesion and proliferation of rat cranial osteoblasts in the scaffold and promote osteogenic differentiation of the cells. Bao and colleagues [17] prepared SMP scaffolds containing molten salt templates using polycaprolactone (PCL) diacrylate. Subsequently, human osteoblasts were cultured in the scaffolds coated with polydopamine, and it was found that the cells could adhere and proliferate well in the scaffolds, and the expression of osteogenic genes and the deposition of extracellular matrix were also increased. Later, water-responsive PBF scaffolds were fabricated and used to culture rat osteoblasts, and the porous structure with a pore size of 75–150 μm was found to support cell adhesion, maintain cell activity, and promote intracellular alkaline phosphatase (ALP) activity. For the scaffold itself, the thermal properties, mechanical properties, water-responsive shape memory effect, and degradation properties were tested in vitro. The results showed that the PBF scaffold exhibited good water-responsive shape memory properties as it quickly returned to its original shape after 3 min in water and also exhibited good biodegradability due to the ester bonds in the scaffold. In addition, it was noted that PBF could be easily functionalized due to its abundant hydroxyl and alkene groups and, therefore, can carry cytokines and release them slowly [30]. In addition to primary osteoblasts, the pre-osteoblasts cell line MCET3-E1 has been shown to remain viable in polyurethane/hydroxyapatite (HA) SMP foams and invade the interior of scaffolds [56].

#### 4.1.2. Effect of SMPs on BMSCs

Since osteoblasts are mainly differentiated from BMSCs, they have been chosen in several studies to validate the potential of SMP in bone tissue engineering. For example, PD-PCLDA foam was obtained by making SMP foam from poly(ε)caprolactone diacrylate followed by a polydopamine coating. In the absence of osteogenic induction, h-BMSCs can still spontaneously differentiate into osteoblasts in this PD-PCLDA foam. So, the PD-PCLDA foam was shown to have the intrinsic ability to thus confirm that the PD-PCLDA foam has the intrinsic ability to promote osteogenesis of h-BMSC and could potentially become a “self-fitting” scaffold for the treatment of severe bone defects as it can recruit progenitor cells and promote osteoinduction [57]. With the development of biotechnology, BMSCs were applied in various optimized SMP composite scaffolds. For example, in Zhang et al.’s study, rabbit-derived BMSCs cultured on a programmable bone screw made of SMPU, HA, and arginine glycyl aspartate, and modified with arginylglycylaspartic acid (RGD) peptide, were also able to undergo good adhesion, proliferation, and osteogenic differentiation in the scaffold [58]. Based on the fact that PCL is not hydrophilic enough, Huang [59] fabricated porous SMP-HA scaffolds containing three components, in which poly (e-caprolactone)-diols (PCL-diols) were used as a shape memory component, dextran was added to the scaffold as a hydrophilic component to compensate for the lack of hydrophilicity of PC. HA, as a mineralized component, was attached to the inner surface of the scaffold to enhance its mechanical properties. Subsequently, BMSCs isolated from Sprague Dawley (SD) rats were cultured in SMP-HA scaffolds. BMSCs could attach to the inner surface of SMP-HA scaffolds, grow and spread on the surface, and differentiate toward osteoblasts. The biodegradation properties were also investigated in vivo, and the results showed that the biodegradation was completed about six months after implantation. Moreover, the angiogenesis test showed that the 80% porosity and 400–500 μm size of the pore structure resulted in good angiogenesis at the implantation site. Therefore, the SPM-HA scaffold has demonstrated its advantages in mechanical properties, bioactivity, and osteoconductivity to a certain extent. However, it is essential to note that the in vivo study was performed by implanting the scaffold subcutaneously in rats and has not been tested in bone tissue. In addition, mouse BMSCs could differentiate into osteoblasts without osteogenic induction in a PLLA-PHBV ultrafine composite scaffold made from poly(3-hydroxybutyrate-co-3-hydroxyvalerate) (PHBV) and PLLA by electrostatic spinning. Meanwhile, this study indicates that PLLA-PHBV ultrafine composite fibers can be used to construct 3D scaffolds in the future, which is expected to optimize further the role of SMP scaffolds in bone tissue engineering [60].

#### 4.1.3. Effect of SMPs on h-MSCs and C2C12 in SMPs

Previous cellular studies have demonstrated the ability of SMP scaffolds to support osteoblasts adhesion, proliferation, and osteogenic differentiation. However, none of these studies examined the effect of programmed shape change on the behavior of cells implanted in SMP scaffolds. Moreover, in addition to osteoblasts and BMSCs, the osteogenic differentiation ability of cells with stem cell properties, such as human adipose stem cells in SMP scaffolds, can also reflect the potential of SMP scaffolds in bone tissue engineering to some extent, as they can differentiate into osteoblasts in osteogenic induction medium. Therefore, Tseng and colleagues [61] fabricated SMP foam and SMP fiber scaffolds using acrylate and thermoplastic polyurethane (TPU) as the primary materials, respectively. Human adipose-derived stem cells (hASCs) were inoculated into the SMP scaffold without triggering shape memory and placed at 30 °C for five days for osteogenesis induction. The scaffolds were then placed at 37 °C to transition the foam scaffold from a temporary compressed pore structure to a permanent open pore structure, and the fiber scaffold from a temporary unaligned structure to a permanent uniaxially aligned structure, and the cells were continued to be cultured in these scaffolds for 23 days. The results showed that hASCs could still differentiate into osteoblasts in the scaffolds and were not affected during scaffold deformation. So, we can see that osteogenic differentiation of hASCs in two different types of programmable SMP scaffolds—foam scaffolds and fiber scaffolds—is based on changes in internal structure triggered by shape memory under cytocompatible conditions and that the feasibility of using shape memory scaffolds in a cell-based strategy to treat bone defects is promising. It has also been shown that the shape recovery properties of TPU can be tuned and optimized, for example, by employing a microcellular foaming process (MCP) method [62]. Therefore, it will be possible to subsequently apply the optimized TPU in animal studies to optimize their use strategies. In addition, it has been proposed to use ESMP obtained by synthesizing SMP from star polylactic acid followed by introducing a 5% wt electroactive aniline trimer (AT) fragment to culture C2C12 cells. Functional studies have revealed that ESMP can significantly promote the proliferation and differentiation of C2C12 cells while satisfying mechanical properties such as tensile strengths and Young’s modulus values, as well as biodegradable, biocompatible, and hydrophilic properties required in bone tissue engineering [63]. However, the osteogenic differentiation ability of C2C12 in this ESMP scaffold has not been verified yet. Since C2C12 myoblast cells are generally considered as muscle progenitor cells, some studies have now used them to explore the application of ESMP scaffolds synthesized by PCL3000-5AT in muscle tissue engineering and found that this ESMP showed a promoting effect on C2C12 adhesion, proliferation, and myogenic differentiation. But it should be noted that in vitro degradation rate tests showed that more than 80% of the weight of PCL3000-5AT-synthesized ESMP was lost in less than 2 days [64]. This excessively rapid rate of degradation can lead to inadequate mechanical support during bone regeneration, so ESMP synthesized by PCL3000-5AT may be more suitable for promoting soft tissue regeneration and repair. At the same time, there are limitations to its use in the implantation of load-bearing bones.

The SMP scaffolds used in all of the above cellular experiments were shown to support cell adhesion, proliferation, and osteogenic differentiation activities in vitro. Therefore, we can tentatively speculate that SMP scaffolds can be localized in the areas of bone defects and support the use of SMPs for the treatment of bone defects in vivo due to their properties, such as osteoconductivity and bioactivity? Here, we need to note that the implantation of scaffolds in the body requires the mobilization of cells associated with bone formation and may also adversely affect other surrounding tissues and cells. Therefore, the natural bone repair effect in vivo needs to be verified in animal models.

### 4.2. Application of SMP in Craniomaxillofacial Defects

In contrast to other parts of the bone tissue in the organism, the craniomaxillofacial (CMF) bone is generally unloaded, and its defects can seriously affect our appearance and health. Therefore, in vivo studies on SMPs in bone tissue engineering were first tested in animal models of CMF defects. For example, during the same period, a study applied PCL-HA scaffolds equipped with and without BMP-2 to in vitro cultures of BMSCs and in vivo implantation of mandibular bone defect in New Zealand White rabbits, respectively. First, the BMP-2-loaded SMP scaffold was placed in water at 37 °C to restore its shape and produce a porous structure with a porosity of 90.6 ± 5.2% and a pore size of 160 μm within 60 s. The scaffold was then used to culture BMSCs in vitro, and the MTT test demonstrated that the scaffold had good cytocompatibility allowing cells to penetrate inside the scaffold and proliferate and grow. Second, in vivo studies were performed in New Zealand rabbits with a 15 × 10 mm^2^ critical size cortical bone defect in both mandibles, followed by implantation of PCL-HA scaffold with BMP-2 and PCL-HA scaffold without BMP-2, and the animals were executed after eight weeks of postoperative micro-CT and histological examination. Unlike scaffolds that take only 60 s to recover the shape when triggered in vitro, in vivo experiments revealed that it took 10 min for the scaffold to return to a fixed shape after implantation into the mandibular bone defect, which bought more time for flexible adjustment when the scaffolds were implanted into the bone defect. Moreover, the release of BMP-2 could reach 65% of the total weight during the first 15 h after implantation, and then the release slowed down. After eight weeks of implantation treatment, the BMP-2-loaded SMP scaffold significantly promoted new bone formation in the defect area of the rabbit mandible compared with the BMP-2-unloaded SMP scaffold, indicating that the combination of BMP-2 and SMP scaffolds significantly promoted new bone formation in the rat mandible [65].

The subsequent studies began to focus on optimizing SMP properties to enhance their mechanical and biodegradable properties for applications in bone tissue engineering. For example, based on the characteristics of the SMP formed by crosslinking poly(ε-caprolactone) diacrylate (PCL-DA), a second polymer component, PLLA, was added to enhance the mechanical properties and degradation rate of the PCL-DA scaffold [66]. Subsequently, PCL-PLLA semi-IPN scaffolds prepared based on PCL-DA and PLLA at different weight ratios were found to maintain shape memory behavior and exhibit tunable accelerated degradation rates, as well as stiffness and strength of scaffolds [67]. Moreover, the performance of the PCL-PLLA semi-IPNs made by the SCPL method [24] was further optimized after adjusting the average degree of polymerization of PCL-DA (*n* = 25, 45) while adjusting the different weight ratios of PCL-DA: PLLA. This was shown by the fact that adjusting the annealing temperature could change the average pore size and porosity, and all PCL-PLLA semi-IPNs exhibited large porosity and interconnectivity of pores. When PCL-PLLA semi-IPNs were made with PCL-DA (*n* = 25) and PCL: PLLA weight percentages of 75:25 and 60:40, respectively, the modulus increased by ~62%, along with a slight increase in scaffold strength compared to the PCL-DA (*n* = 25) control. Thus, it is clear that thermal properties, shape memory behavior, degradation rate, and mechanical properties can be integrated to form a comprehensive SMP scaffold with a defective “fit” that meets the requirements for CMF defect repair [68]. However, such a scaffold is an obvious limitation: the minimum annealing temperature is 85 °C, and too high an annealing temperature can damage the tissue. Therefore, Pfau et al. [69] prepared PCL-PLLA semi-IPN scaffolds by transitioning from polymers with a linear structure to polymers with a star structure to optimize the SMP scaffold performance and prepared six of them: linear-PCL-DA (*LPCL*), star-PCL-TA (*SPCL*), *linear-PCL-DA/linear-PLLA*(*L/L*), *linear-PCL-DA/star-PLLA*(*L/S*), *star-PCL-DA/linear-PLLA*(*S/L*), and *star-PCL-DA/star-PLLA*(*S/S*). The average porosity of all scaffolds was 60%, and the pore size was ~220 μm. The S/S semi-IPN scaffold was possibly more favorable for application in the repair of CMF defects because it showed desirable properties in shape memory function, degradation rate, improved osseointegration, and mechanical stability. Nevertheless, such a composite scaffold has not been validated by any in vitro or in vivo experiments. Subsequently, Arabiyat et al. [27] demonstrated for the first time through cellular experiments that PCL-DA/PLLA semi-IPN scaffolds showed more vigorous intrinsic osteoinductive activity compared to PCL-DA. Moreover, PCL-DA/PLLA semi-IPN scaffolds maintained a porosity of approximately 65% and a pore size of 180–190 μm after PD coating, further enhancing the osteogenic capacity of SMPs. Thus, this study further indicates the superior therapeutic potential of PCL-DA/PLLA semi-IPN scaffolds coated with PD in craniomandibular defects.

Many studies have characterized the mechanical properties of SMP itself concerning parameters such as elastic modulus. However, little attention has been paid to the mechanical contact properties between the SMP implant and the edge of the bone defect. In a recent study, a mechanical test fixture and paired alignment components were fabricated by 3D printing using Acrylonitrile butadiene styrene (ABS). The mechanical push-out bar was vertically aligned with the bone defect in cranial defect models of rats and New Zealand rabbits, respectively. It resulted in the development of a push-out test to assess the strength between the implants and the edge of the bone defect [70]. Michaela and colleagues [71] then affirmed the role of PCL-DA SMP scaffold for bone repair in non-critical zone size cranial defects in 6-month-old New Zealand rabbits. Unlike other studies, this study only tested the scaffold’s performance without adding any growth factors, cells, and coatings. PCL-DA scaffolds with a porosity of 70% and a mean pore size of 220 μm and a diameter of 9 mm were immersed in warm saline at 55 °C for 30–60 s and implanted in a calvarial defects area of 8 mm in diameter. This design provided good contact at the scaffold-bone tissue interface and simultaneously allowed the scaffold to mechanically stimulate the bone around the defect area with compressive stress to promote bone formation after shape recovery, as the scaffold diameter is slightly larger than the defect diameter. The push-out tests conclusively confirmed that the scaffold had ideal osseointegration and stability. After 16 weeks of implantation, vascular, fibrous, and bone tissue were found to have invaded the interior of the SMP scaffold, and push-out tests ultimately confirmed that the scaffold had ideal osseointegration and stability. In addition, scaffolds of 1 mm and 2 mm thickness were designed to test the required scaffold thickness in rat calvarial defects repair. It was found that the scaffold of 2 mm thickness was more suitable for bone repair because it was closer to the thickness of the rat cranium itself, and it can be inferred that the thickness of the SMP scaffold is also a critical factor that should be considered in the subsequent studies on the repair of human craniomandibular bone.

### 4.3. Application of SMP in Limb Bone Defects

Compared to the repair of CMF defects, limb bones, especially weight-bearing bones, require more mechanical properties of the SMP scaffolds, which have only been researched in a few studies. One study established a femoral defect model by removing 4 mm of bone from the mid-femur in 12-week-old C57BL/6J female mice. Then SMP grafts and SMP sleeves were prepared by electrostatic spinning using TPU and implanted into the defect area to fill the gap and assist in fixing the graft, respectively, because SMP grafts can be expanded to fill the bone defect. In contrast, the SMP sleeves can contract around the femur at 45 °C in saline. The results showed that the SMP grafts could integrate with the natural bone and maintain the stability of the defect area after 12 weeks of implantation, although no significant new bone production was observed, and the SMP sleeves could provide additional torsional stability to the defect. Based on the pore size required for bone formation and vascular invasion, perhaps a further increase in pore size from the current pore size of 58 μm may accelerate the progress of bone defect recovery by improving bone formation and hematologic reconstruction. In conclusion, it was found that the mechanical properties of these SMP grafts and sleeves were sufficient to maintain defect stability for the animal model. This application of shape memory technology to an artificial bone graft substitute and an adjunctive stabilization device is expected to enhance the use of SMP scaffolds for load-bearing bone defect repair. However, whether the SMP sleeve damages the periosteum needs to be evaluated in a follow-up study, as it tightly wraps around the periosteum when contracted [72].

In another study, a rabbit femoral defect model was established by drilling out bone tissue of 4.5 mm in diameter and 6 mm in height from the femoral talar groove of New Zealand White rabbits. Polyurethane/HA SMP foam prepared by the gas foaming method had 60% porosity, an average pore size of 670 μm, and 99% porous interconnectivity. After triggering the shape memory function in saline at 40 °C, this SMP foam expanded rapidly to fill the femoral defect within 60 s. Moreover, bone mineralization around the SMP foam spread to the interior and gradually increased at weeks 4, 8, and 12 after implantation, and the percentage of repaired bone defects in the SMP foam group was as high as 46% at 12 weeks after implantation, significantly higher than the 24% of bone repair in the control group. In addition, HE staining also revealed a significant increase in the number of neovascular buds and osteoblasts in the SMP foam group, which significantly promoted vascularization and bone remodeling [56]. However, this study did not test the mechanical properties of this SMP, and no significant degradation of the SMP was observed over 12 weeks in vivo. Therefore, the biodegradation properties of SMP foam and the toxicity of degradation products to tissues need to be evaluated in the future.

The above two studies have investigated the repair of defects in the middle femur using different SMPs. However, the repair potential of SMP at the junction of the cortical and trabecular bone of the femur, especially the condylar defect, has not been investigated. Therefore, Zhang et al. [58] used a programmable SMPC screw made of SMPU/HA/RGD composite first to validate its ability to promote BMSCs survival, proliferation, and differentiation in vitro and then established an animal model of a femoral condylar defect by drilling a hole of approximately 3 mm in diameter in the medial femoral condyle of New Zealand rabbits from the inside out. The SMPC screw implanted into the defective bone rapidly filled the defect within 20 s and provided continuous mechanical support. The results showed a significant increase in bone formation after three months of implantable treatment.

Based on the current results of all cellular and animal experiments on the application of SMP materials in bone tissue engineering (as shown in Table 1), we can find that SMPs have a reasonably good therapeutic potential, and several of the different SMPs even seem to perform well in the repair of load-bearing bone defects. Nevertheless, first of all, we must note that the therapeutic period of SMPs implantation in animals in the three studies mentioned above lasted only 12 weeks, so it is necessary to develop longer-term animal models to study the degradation of the scaffold in vivo and the long-term therapeutic effects. Secondly, there is no doubt that the mechanical properties required of SMPs to repair load-bearing bone defects in humans are much higher than those required for application in these small animal models. Therefore, although SMPs have excellent potential for bone tissue engineering, they are still far from being transitioned to clinical applications.

## 5. Strategies and Challenges for Optimizing the Application of SMPs in Bone Tissue Engineering

### 5.1. Promote Bone Formation with Emphasis on Bone Angiogenesis

**Figure 2:**
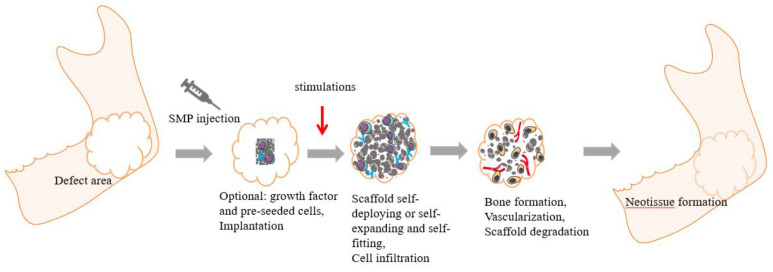
General schematic diagram of SMP loaded growth factors applied in bone tissue engineering. Based on simulations, a highly porous scaffold carrying optionally loaded growth factors and cells is implanted in the bone defect area to trigger its shape memory property and fill in the defect area. Subsequently, the cells and growth factors affect the scaffold to promote bone formation and angiogenesis. Eventually, the scaffold is degraded and replaced with neotissue [13].

Bone angiogenesis and bone formation are two biological processes that coincide in bone growth and development and bone regeneration and reconstruction. Regeneration of blood vascular is essential for bone formation, a complex process involving mobilization, chemotaxis, adhesion, proliferation, and differentiation of vascular endothelial progenitor cells (EPCs) [74]. There are three general strategies to enhance angiogenesis in tissue-engineered scaffolds. The first is the integration of various pro-angiogenic regulators, e.g., RGD, vascular endothelial growth factor (VEGF), and mechanical stimulation that induces vascular growth in surrounding tissues or recruit EPCs [75]. The second is the formation of blood vascular in vitro by cellular engineering [76]. Moreover, the third strategy is prevascularization in vivo before transplantation into damaged tissues [77]. In studies of bone disease treatment, the first approach is more often chosen for research, and the generation of pro-angiogenic effects based on this approach usually relies on delivery vehicles to transport pro-angiogenic factors to the damage or defect site in the tissue. For example, dextran can be chemically engineered into scaffolds such as tubes or spheres, thus serving as a tissue regeneration device and a cell therapy vehicle [78]. However, it usually requires chemical modification to introduce functional affinity sites, as in the case of dextran, which has been shown to promote angiogenesis by binding to PCL to form polymers embedded in hydrogels to encapsulate specific vascular growth factors used for therapeutic neovascularization [79]. A few studies have obtained the desired therapeutic effect in treating bone diseases by exogenous input of angiogenic factors such as VEGF and osteogenic factors such as BMPs or in combination with stem cell therapy. Therefore, angiogenic and osteogenic factors encapsulated in the porous structure of the scaffold may be a new idea for future research, as this could contribute to the continued growth and invasion of new bone and vascular tissue (as shown in Figure 2). It has also been found that SMPs scaffolds located in deep tissues can contract in volume when subjected to non-contact thermal stimulation to induce the release of substances within the scaffold if they are combined with substances such as infrared light photosensitizers, this proves that carrying bioactive factors such as various proteins in SMPs composite scaffolds are expected to achieve on-demand or sustained release of these factors in specific spaces and at specific times [80,81]. Similarly, 4D printing techniques currently being investigated may also enable scaffolds to undergo dynamic changes in the tissue [82]. However, the mass of bioactive regulators to be added, the ratio of bioactive regulators to be coordinated, and the rate of bioactive regulator release in vivo has yet to be investigated in animal or human experiments.

### 5.2. Increase the Gap between Bone and Periosteum through SMP to Promote Intramembranous Osteogenesis

The periosteum is rich in vascular and nerve tissue and has many bone progenitor cells that can develop into mature osteocytes and eventually become bone tissue, so intramembranous osteogenesis is an essential way of bone formation [83]. Recent studies have invented interventions for periosteal distraction osteogenesis based on intramembranous osteogenesis, which means that additional external forces are applied, or the periosteum is slightly stripped from the bone surface, or a scaffold is implanted between the bone and the periosteum to form a subperiosteal gap between the surface of the periosteum and the bone, thus inducing the production of new bone [84,85,86]. Therefore, He et al. [73] used this principle to co-implant a titanium implant and an SMP scaffold made of PCL-HA into the rabbit mandible, which recovered its shape after about 1 min in PBS at 37 °C and was able to form a small gap between the periosteum and the bone. The final results showed that the SMP scaffold was gradually replaced by newly formed bone at 12 weeks after implantation and that the stability of the implant accompanying the scaffold implant was increased. However, the degradation rate of the SMP scaffold in this study was only tested in vitro, and in vivo degradation needs to be further observed. In conclusion, although the use of SMP based on intramembranous distraction osteogenesis is helpful for bone formation, there are still some challenges, first of all, the treatment period is too long and the periosteum may be damaged if not performed properly, as the degree of the distraction of the periosteum is difficult to control.

## 6. Summary and Outlook

SMP scaffolds synthesized from materials including PCL, PLA, PU, and PLLA have recently attracted much attention in tissue engineering. PCL has been most frequently studied in bone tissue engineering based on its easy availability, reasonable cost, and excellent performance. It is also commonly used in bone tissue engineering applications by blending with other materials as composite scaffolds. These SMP scaffolds have shown good application prospects in bone tissue engineering. However, it is worth noting that the current potential of SMP scaffolds in bone tissue engineering has only been studied in cellular experiments and animal bone defect models. Moreover, most of the studies still focus on the repair of irregular defects in CMF, and only a few scholars have observed the repair effect of SMP on limb bone defects in animal models, while the use of SMP in human bone tissue has not been clinically reported at all.

Nevertheless, the unique shape memory ability and biodegradable properties of SMP scaffolds are irreplaceable by other materials. Therefore, optimizing the performance of SMP scaffolds prepared from existing materials in various ways is a possible direction for future research. With the advancement of materials science, using multiple shape memory materials in combination has become a new trend in current research, as it can simultaneously meet several needs for bone tissue engineering. However, the combination of which materials and by what methods to prepare SMP composite scaffolds that maximally meet the needs of bone tissue regeneration and repair is a direction that scientists are trying to explore. First, in addition to comparing which materials to blend to synthesize SMP scaffolds that perform better, it may be possible to try to develop new materials with more excellent utility. Second, the latest research has begun to focus on applying 4D printing technology in bone tissue engineering. Combining 4D printing technology with the currently mature methods of preparing SMP scaffolds (such as the SCPL method, electrospinning method, and gas foaming method) for SMP preparation may achieve a dynamic change in the scaffold in the tissue. In addition, using SMP scaffolds in combination with biological agents to promote bone defect repair by simultaneously promoting bone angiogenesis and bone formation is also highly feasible. In conclusion, SMP has good applicability in bone tissue engineering and will eventually lead to the clinical application of SMP materials in repairing bone defects.

## Figures and Tables

**Table 1:** Application of SMPs in bone tissue engineering.

SMP Material	Cell/Tissue	Species	Effects on Bone Engineering	Disadvantage	References
PDLLA-*co*-TMC	Calvarial osteoblasts	Rat	Support adhesion and proliferation of osteoblasts and functionally promote the expression of alkaline phosphatases and mineral deposition	-	[55]
PCL, polydopamine-coated scaffolds	Osteoblasts	Human	Support osteoblasts adhesion, proliferation, and osteogenic gene expression/extracellular matrix deposition	-	[17]
PCL-HA	BMSCs	Rat	Promote adhesion, proliferation, and osteogenic differentiation of BMSCs, and promote angiogenesis		[59]
PCL-DA	-	-	-	-	[24]
PCL-DA	Skull	Rabbit	Promote osseointegration at the scaffold/defect interface	-	[71]
PD-PCLDA	h-MSCs	Human	Promote the osteogenic differentiation of h-MSCs	-	[57]
tBA-BA and TPU	ASCs	Human	Both foam scaffolds and fiber scaffolds promote osteogenic differentiation of h-ASCs	-	[61]
PCL-PLLA	-	-	To a certain extent, the thermal properties, shape memory behavior, degradation rate, and mechanical properties meet the requirements for repairing irregular cranial bone defects	-	[68]
PCL-HA	MC3T3-E1/Femur	Rabbit	Support MCET3-E1 cell infiltration and growth, promote angiogenesis and bone formation in the femoral defects	Slow degradation rate	[56]
BMP-loaded cPCL-HA	BMSCs/mandibular	Rabbit	Support the growth of BMSCs in vitro and promote the healing of mandibular defects in rabbits in vivo	Slow degradation rate	[65]
PBF	Calvarial osteoblasts	Rat	Promote osteoblast adhesion, viability, and alkaline phosphatase activity	-	[30]
SMPU-HA-RGD	BMSCs	Rabbit	Support survival, proliferation, and osteogenic differentiation of BMSCs in vitro; promote bone formation in the femoral defect in vivo	The modulus is not sufficient to replace metal screws for cortical bone repair	[58]
PCL-HA	BMSCs/mandibular	Rabbit	Increase the stability of titanium implants and promote bone formation	Lack of examination of degradation rate in vivo	[73]
PLLA-PHBV	BMSCs	Mouse	Promote osteogenic differentiation of BMSCs	-	[60]
PCL-DA/PLLA semi-IPN	H-MSCs	Human	Promote osteogenic differentiation of h-MSCs	-	[27]

-: not mentioned.

## Data Availability

No new data were created or analyzed in this study. Data sharing is not applicable to this article.
